# Meaning in Life: Exploring the Potential of Mythological Narratives in Contemporary Life

**DOI:** 10.1007/s11013-025-09933-4

**Published:** 2025-08-08

**Authors:** Nader Abazari, Suvi-Maria Saarelainen

**Affiliations:** School of Theology, Philosophical Faculty, University of Eastern Finland, Joensuu, Finland

**Keywords:** Meaning, Meaning-making, Existential concern, Myth, Comparative mythology

## Abstract

In this article, we address the potential of mythological narratives to contribute to understanding meaning in life. Drawing on contemporary theories concerning meaning in life including the existential psychotherapeutic approach, discrepancy-based model and hierarchical meaning-making model, we highlight key principles of the meaning-making process that are reflected also in world mythological narratives. These common principles include encountering a profoundly impactful event, dissatisfaction with the current state, meaning-making attempts, and providing a vision of the desired state. Consequently, by integrating insights from psychology and anthropology, we propose that myths not only continue to hold enduring relevance in contemporary life but also possess considerable potential as frameworks for enriching knowledge on what makes life meaningful and pathways that help individuals experience meaning in their lives.

## Introduction

The central human motivation to find meaning (see Frankl, 1963) pertains to the search for a sense that our lives matter, are worth living, and amount to more than the mere accumulation of days (Steger, 2012). Distinguished from the more philosophical concept of the meaning of life, which concerns the ultimate purpose of human existence and the universe as a whole (Debats, 1998, p. 359), the concept of meaning in life focuses on the value of an individual’s personal existence (Metz, 2013, p. 17). It is understood as a multidimensional construct, grounded in the subjective evaluation of life as either meaningful or devoid of meaning (Schnell, 2009). Meaning in life not only enables individuals to experience their lives as significant, purposeful, coherent, and embedded within a sense of belonging (e.g. Park, 2010; Schnell, 2020), but also influences psychological well-being, life satisfaction, and both mental and physical health (see Czekierda et al., 2017; Schnell et al., 2018; Ojalammi et al., 2024).

Given that humans naturally organize their experiences into meaningful patterns (Koffka, 2013), and that meaning is not inherent in life per se (Park, 2010) but must be actively created by individuals within cultural contexts (Schnell, 2020), it is evident that long before scientific research began systematically exploring meaning in life (e.g., Frankl, 1963; King & Hicks, 2021; Park & Folkman, 1997; Schnell, 2009; Steger et al., 2006; Wolf et al., 2012; Yalom, 1980), humanity addressed the quest for meaning through shared cultural heritage (Gant, 2022). For instance, the heroic journey depicted in world myths symbolizes the quest for spiritual fulfillment, a sense of meaning, and a creatively inspired life (Campbell, 1993b, p. 13).

While the everyday use of the word “myth” often refers to a false story or belief (Leeming, 2003), we use the term in this paper to refer to narratives with profound cultural significance that help humans make sense of their existence (see Krader, 2021). In our usage, myth represents narratives that reflect shared perceptions of reality (Campbell & Kudler, 2004), providing a wide range of functions, such as helping humans understand the origins of humanity and the world (Geertz, 1972), justifying rituals associated with daily life (Wagner, 2016), and presenting moral guidance on desirable and undesirable behaviors (Jacopin, 1987). In this context, myth serves to validate and sustain collective notions of right and wrong, as well as what is considered proper or improper (Campbell & Kudler, 2004). Viewed more holistically, myths have functioned to situate life within a broader perspective and to explore underlying patterns, meanings, and values (Armstrong, 2006), helping humans find their place in the wider cosmos (Thompson & Schrempp, 2020), much like how a sense of meaning in life enables individuals to find their place within the chaos of the world (see Baumeister, 1991).

Current understanding of meaning in life indicates that individuals experience their personal existence within the framework of an environment that both precedes and outlasts them (Durkheim & Bellah, 1973, pp. 35–38). In other words, their sense of meaning draws upon beliefs, values, and attitudes inherited from the surrounding cultural context (Steger et al., 2006, p. 383). Furthermore, it is widely recognized that humans are constantly engaged in the meaning-making process in life (e.g., Czekierda et al., 2017; Park, 2010), and that the ideas embedded in myths remain relevant to contemporary life (e.g. Campbell & Kudler, 2004; Gant, 2022; Lévi-Strauss, 1955). Drawing on theoretical frameworks addressing meaning in life (incl. Park & Folkman, 1997; Schnell, 2009; Yalom, 1980) and examining mythological examples, we explore the potential of mythological narratives to contribute to knowledge on humanity’s meaning-making process in today’s world.

## Meaning in Life: Conceptual Parallels Between Contemporary Theories and Mythological Narratives

To explore the potential of myths to contribute to understanding of the meaning-making process, we address the common elements of three theories concerning meaning in life: the existential psychotherapeutic approach (Yalom, 1980), discrepancy-based model (Park & Folkman, 1997) and hierarchical meaning-making model (Schnell, 2009). These theories are more psychological than philosophical in nature (Steger et al., 2006), have been applied in mental health studies (Branitsky, 2024), and are characterized by a less subjective approach (Schnell, 2020). Furthermore, mythological examples from the Finnish folklore Kalevala, Mesopotamian epic of Gilgamesh, Persian literature the Shahnameh, Sufi literary tradition the Conference of the Birds, Greek mythology, and the Sanskrit epics of ancient India are explored. These myths, originating from diverse cultural contexts, geographical regions, and worldviews (see Leeming, 2005), depict protagonists striving to create meaning in their existence (see May, 1991).

First, all three theories of meaning in life link the onset of meaning-making to an encounter with a profoundly impactful event or a turning point. These include irreversible dilemmas and confrontations with mortality (Yalom, 1980), external events such as loss or illness (Czekierda et al., 2017; Park, 2022), and internal conflicts (Schnell, 2009). Similarly, world myths are abundant with narratives depicting challenging and perilous journeys undertaken in the pursuit of a meaningful life (Campbell, 1993b, p. 27), often triggered by a pivotal event marking a turning point in the protagonist’s path. For instance, in the Kalevala, Väinämöinen’s journey to find the Sampo (a symbol of endless prosperity) is driven by the pressing needs of his people. In the epic of Gilgamesh, the hero embarks on his long journey to reach immortality after witnessing the death of his closest companion and grappling with the suffering of loss. Likewise, in the Shahnameh, Rostam’s Seven Labors commence when the Shah is captured by the White Demon. Also, the Conference of the Birds depicts the search for the Simorgh (a symbol of divine truth), which unfolds after the city is plunged into darkness. Each journey highlights the profound impact of a transformative event, setting the protagonist on a path of goal, growth, self-discovery, and meaning (see Campbell, 1993b; Armstrong, 2006; Masoudifard, 2013).

Second, all three theories predict that individuals will experience dissatisfaction with their current state following an impactful event. These consist of an experience of existential anxiety (Yalom, 1980), a discrepancy between global meaning and situational meaning (Park, 2010), and a perception of life as meaningless (Schnell, 2009). In this regard, if the gap between the current state and the desired state is minor, no effort is made to create meaning (e.g. Czekierda et al., 2017). However, when the gap becomes significant enough to cause distress, individuals are compelled to actively engage in meaning-making (Park, 2022; Schnell, 2020). This pattern is also evident in mythological narratives. For instance, in the human creation myth in the Kalevala, Väinämöinen passively waits for ten years in the cramped and dark confines of Ilmatar’s womb. Only after becoming deeply weary of his restricted state, through persistent physical movements, he frees himself and experiences freedom. Similarly, in the Shahnameh, Kaveh the Blacksmith initially tolerates Zahhak’s tyranny, watching passively as young men are killed to feed the snakes on Zahhak’s shoulders. However, when his own sons’ lives are at risk, he rises to challenge Zahhak, initiating a rebellion that becomes a symbol of resistance. These examples underscore the argument that individuals are often driven to act in the meaning-making process when they recognize that their existing cognitive and behavioral patterns are no longer effective and that a pressing need for change has arisen (Park, 2010; Schnell, 2020).

Third, each of the theories conceptualizes meaning-making attempts as a fundamental principle. This process encompasses the use of defense mechanisms and coping strategies (see Yalom, 1980), the dynamics of accommodation and assimilation (see Park, 2010), and adherence to sources of meaning (see Schnell, 2009). Likewise, the meaning-making efforts of mythological characters encompass a diverse range of actions. For instance, in the Shahnameh, Arash sacrifices his life by shooting an arrow over an extraordinary distance to protect his homeland. In Greek mythology, Odysseus devises the deception of the Trojan Horse, enabling his people to conquer Troy. Similarly, in another Greek myth Prometheus, in his desire to aid humanity, deceives the gods by stealing fire and gifting it to humans. Surprisingly, although the research literature often identifies loss as a disruptive event that challenges meaning in life (e.g., Park, 2010; Vähäkangas et al., 2022), the Conference of the Birds portrays loss such as the loss of possessions, beauty, and knowledge as a transformative means to achieve the ultimate goal. This perspective highlights the deeply subjective and multifaceted nature of meaning-making in life (see Wolf et al., 2012).

Fourth, providing a vision of the desired state is another key principle commonly emphasized by theorists. In Yalom’s framework, this desired state is characterized by the alleviation of existential anxiety; in Park’s model, it involves reducing the discrepancy between global meaning and situational meaning; and in Schnell’s theory, it is defined as perceiving life as meaningful. Like the current theoretical frameworks, mythological narratives often convey these desired states either directly or through symbolism. For instance, the desired state for Väinämöinen in the Kalevala is to obtain the Sampo, and for Prometheus it is to aid humanity by giving them fire. Also, in the Sanskrit epic of ancient India, Savitri’s potential death when confronting the God of death signifies immortality through the act of saving her husband’s life (see Campbell, 1993a). Additionally, the birds in the Conference of the Birds perceived the loss of their possessions as a necessary step toward achieving the ultimate goal of finding the Simorgh (Masoudifard, 2013).

Assuming that conceptual parallels in the meaning-making process such as encountering a profoundly impactful event, dissatisfaction with the current state, meaning-making attempts, and providing a vision of desired state can be identified between contemporary theories and mythological narratives, we now turn to potential explanations that may help account for the continued relevance of mythological narratives in the contemporary world.

## The Enduring Relevance of Mythological Narratives in Today’s World

Psychological insights offer both theoretical frameworks and empirical evidence to explain the enduring relevance of mythological narratives in the contemporary world. For instance, Freud (2012a, b) regarded an individual's personal history as intrinsically connected to the broader history of humanity. Within this framework, whether events are objectively real or merely perceived as real by an individual or group (see May, 1991), past experiences can leave enduring imprints on the psyche, just as collective experiences in human history can shape the cultural norms and collective mentality of a society. Developing this idea further, Jung argued that myth reflects the structure and dynamics of the human mind more than it represents the external world (Segal, 2003), theorizing the collective unconscious as the repository of universal, heritable elements shared by all humans (see Jung, 2014). In this vein, cognitive psychological study has demonstrated that inherited knowledge stored in human memory can be activated and subsequently influence behavior (e.g. Escribe & Huet, 2005; Higgins, 1991). This supports the view that myths function as symbolic representations of human history that offer valuable insights for navigating a rapidly changing and unpredictable future (Rabaté, 2013, pp. 172–174).

Moreover, anthropological perspectives provide alternative explanatory frameworks. Lévi-Strauss and Wilcken (2013) argued that, since myths across cultures share underlying structures despite differences in surface details, they might be studied in a manner analogous to language. Following this line of thought, Geertz (1972, 2009) and Turner (1975) proposed that symbols serve as the fundamental units of human communication, creating shared understandings between individuals within specific cultural frameworks. These symbols accumulate within myths to form a system of common symbolic meanings. Further emphasizing the cultural grounding of myth, Boas (1904) highlighted the pluralistic nature of culture, arguing that there are many distinct cultural realities, which in turn shape different interpretations of the symbols inherited in myths and expressed through rituals (Wagner, 2016). Myths, in turn, offer symbolic frameworks for interpreting life’s challenges and proposing culturally meaningful solutions, which are enacted and reinforced through ritual practices (see Malinowski, 1984; Seremetakis, 2017).

In much the same way, Dundes (1969) highlighted the cultural function of myths, and Radin (1929) advocated for a historical–psychological approach to the study of humans, arguing that people understood the world in terms of how it affected them emotionally and practically, and had long expressed these understandings through myths. Reflecting their dynamic nature, myths are thus repeated and transformed over time rather than simply told once, as they address recurring human concerns across generations (see Lévi-Strauss, 1955). In light of this, we can view myths not only as summaries of cultural essence (Witzel, 2012), but also as carriers of symbolic meaning into everyday life through the medium of ritual (Jacopin, 1987), providing general and transmissible understandings, expressed as culturally encoded facts, that individuals encounter and engage with in the specific contexts they live in (see Evans-Pritchard, 2013).

Taken together, these psychological and anthropological perspectives underscore the timeless role of myths in human life, leading us to recognize their enduring relevance in the contemporary world.

## Exploring Meaning in Life Through Mythological Narratives

Drawing on the literature (e.g. Frankl, 1963; King & Hicks, 2021; Park, 2010; Schnell, 2020; Steger et al., 2006), we first find that meaning is not inherently embedded in human existence but must be actively constructed by individuals themselves. Subsequently, these studies highlight that meaning-making does not occur in isolation, nor is it solely shaped by immediate social environments or situational factors; rather, it is profoundly influenced by the broader cultural frameworks within which individuals live. This is further reflected in Baumeister’s (1991) perspective. While acknowledging that meaning is fundamentally personal and subjective in nature, he contends that individuals primarily select from among the meanings made available by their culture.

In light of these considerations, given that the content embedded in myths reflects a rich synthesis of the cultural contexts in which they emerge (e.g. Boas, 1904; Jacopin, 1987; Lévi-Strauss, 1955; Malinowski, 1984; Witzel, 2012), that notable parallels can be identified between contemporary meaning-making theories and mythological narratives, and that myths continue to hold enduring relevance in the contemporary world (e.g. Dundes, 1969; May, 1991; Segal, 2003; Freud, 2012b; Rabaté, 2013; Jung, 2014; Seremetakis, 2017), we propose that mythological narratives, as the collective history of humanity (see Leeming, 2005; Armstrong, 2006), hold significant potential for advancing our understanding of meaning in life. As emphasized by May (1991), myths not only allow individuals to trace the origins of humanity but also offer guidance on what makes life meaningful and how such meaning can be attained.

## Conclusions

Bringing these notions together, we propose that mythological narratives, far from being mere relics of the past, remain deeply relevant in today’s world by offering existential insights and reflecting enduring human concerns about meaning in life through key aspects of the meaning-making process. Moreover, since previous research has highlighted cultural differences in meaning-making processes (e.g. Ojalammi et al., 2024; Steger et al., 2006), we suggest that mythological narratives provide a valuable framework for exploring these variations in cross-cultural investigations.

## Data Availability

No datasets were generated or analysed during the current study.
